# Exploring Racial Differences in the Association of Plasma Biomarkers and *APOE4* Genotype in a Large Memory Clinic Cohort

**DOI:** 10.1002/alz70856_106873

**Published:** 2026-01-10

**Authors:** Xuemei Zeng, Rebecca A Deek, Michel N Nafash, Jeremy M. Gu, Lamia Choity, Tara K Lafferty, Marissa F Farinas, Margaret A Bedison, Rocco B Mercurio, Cristy Matan, Alexandra Gogola, Julia K. Kofler, Dana L Tudorascu, C. Elizabeth Shaaban, Jennifer H Lingler, Tharick A Pascoal, William E Klunk, Victor L. Villemagne, Milos D. Ikonomovic, Sarah B Berman, Robert Sweet, Beth E. Snitz, Ann D Cohen, M. Ilyas Kamboh, Oscar L Lopez, Thomas K Karikari

**Affiliations:** ^1^ University of Pittsburgh School of Medicine, Pittsburgh, PA, USA; ^2^ University of Pittsburgh, Pittsburgh, PA, USA; ^3^ School of Public Health, University of Pittsburgh, Pittsburgh, PA, USA; ^4^ University of Pittsburgh Alzheimer's Disease Research Center (ADRC), Pittsburgh, PA, USA; ^5^ University of Pittsburgh, School of Medicine, Pittsburgh, PA, USA; ^6^ VA Pittsburgh Healthcare System, Pittsburgh, PA, USA; ^7^ Department of Psychiatry and Neurology, University of Pittsburgh, Pittsburgh, PA, USA

## Abstract

**Background:**

The *APOE4* is a major genetic risk factor for Alzheimer's disease (AD) and has been shown to impact plasma AD biomarker levels. We utilized a large memory clinic cohort to assess potential differences in this association race.

**Method:**

Participants at the University of Pittsburgh Alzheimer's Disease Research Center (Pitt‐ADRC) underwent baseline blood collection and cognitive function assessment using the Clinical Dementia Rating (CDR) scale, followed by annual CDR assessments for a median of 3.0 year (IQR 1.9‐5.9). *APOE* genotyping was determined using TaqMan assays. Plasma *p*‐tau181, *p*‐tau217, brain‐derived tau (BD‐tau), GFAP and NfL, were measured using SIMOA assays. Cohen's d and Kaplan‐Meier analysis were employed for statistical inference.

**Result:**

This study included 4,073 participants (59.9% female; 90.2% self‐identified non‐Hispanic White [NHW]), aged 71.9 ± 9.8 years. Both *APOE4* genotype and race were significantly associated with cognitive decline. Black/African American (B/AA) *APOE4* non‐carriers had the most stable cognition over time, with a median stability of 11.3 years, followed by 7.2 years for B/AA carriers. Importantly, NHW showed more cognitive decline than B/AA regardless of *APOE4* carriage, with median stability times of 4.9 and 6.0 years for *APOE4* carriers and non‐carriers, respectively. All biomarkers, except NfL, showed significant *APOE4*‐dependent levels, with effect sizes (carriers/non‐carriers) of 0.176 for *p*‐tau181, 0.361 for *p*‐tau217, 0.118 for BD‐tau, and 0.215 for GFAP. The *APOE4* effect on BD‐tau was insignificant in B/AA, but *p*‐tau217 had a larger effect size in B/AA (0.50) than NHW (0.35). The *p*‐tau217/BD‐tau ratio further highlighted differences between racial groups, with effect sizes of 0.39 for B/AA and 0.07 for NHW.

**Conclusion:**

Racial identity may significantly influence the associations between *APOE4* genotype and plasma biomarkers. This should be considered when applying these biomarkers in diverse populations.

